# Bidirectional causal relational between frailty and mental illness: a two-sample Mendelian randomization study

**DOI:** 10.3389/fpsyt.2024.1397813

**Published:** 2024-06-07

**Authors:** Letian Ma, Zuying Liu, Lijun Fu, Jiaming Fan, Cunlong Kong, Tao Wang, Huilian Bu, Qingying Liu, Jingjing Yuan, Xiaochong Fan

**Affiliations:** ^1^ Department of Pain Medicine, the First Affiliated Hospital of Zhengzhou University, Zhengzhou, China; ^2^ Henan Province International Joint Laboratory of Pain, Cognition and Emotion, Zhengzhou, Henan, China; ^3^ Department of Anesthesiology and Perioperative Medicine, the First Affiliated Hospital of Zhengzhou University, Zhengzhou, China

**Keywords:** frailty index, Mendelian randomization, mental illness, causality, older people

## Abstract

**Background:**

Frailty has been associated with mental illness (MI) observational studies, but the causal relationship between these factors remains uncertain. We aimed to assess the bidirectional causality between frailty and MI by two-sample Mendelian randomization (MR) analyses.

**Methods:**

To investigate the causal relationship among them, summary statistics of frailty index (FI) and six types of MI: anxiety, depression, affective disorder, mania, schizophrenia, and obsessive-compulsive disorder (OCD) were included in this MR study. This MR analysis was performed using inverse variance weighting (IVW), MR-Egger regression, and weighted median. The stability of the results was evaluated using Cochran’s Q test, MR-Egger intercept test, Funnel Plots, and leave-one-out analysis.

**Results:**

Genetic predisposition to FI was significantly associated with increased anxiety (odds ratio [OR] = 1.62, 95% confidence interval [CI] 1.13-2.33, *P* = 8.18E-03), depression (OR = 1.88, 95% CI 1.30-2.71, *P* = 8.21E-04), affective disorder (OR = 1.70, 95% CI 1.28-2.27, *P* = 2.57E-04). However, our study findings do not demonstrate a causal relationship between FI and mania (OR = 1.02, 95% CI 0.99-1.06, *P* = 2.20E-01), schizophrenia (OR = 1.02, 95% CI 0.07-0.86, *P* = 9.28E-01). In particular, although the IVW results suggest a potential causal relationship between FI and OCD (OR = 0.64, 95% CI 0.07-0.86, *P* = 2.85E-02), the directions obtained from the three methods we employed ultimately show inconsistency. Therefore, the result must be interpreted with caution. The results of the reverse MR analysis indicated a statistically significant and causal relationship between anxiety (OR = 1.06, 95% CI 1.01-1.11, *P* = 2.00E-02), depression (OR = 1.14, 95% CI 1.04-1.26, *P* = 7.99E-03), affective disorder (OR = 1.15, 95% CI 1.09-1.21, *P* = 3.39E-07), and schizophrenia (OR = 1.02, 95% CI 1.01-1.04, *P* = 1.70E-03) with FI. However, our findings do not provide support for a link between mania (OR = 1.46, 95% CI 0.79-2.72, *P* = 2.27E-01), OCD (OR = 1.01, 95% CI 1.00-1.02, *P* = 2.11E-01) and an increased risk of FI.

**Conclusion:**

The MR results suggest a potential bidirectional causal relationship between FI and anxiety, depression, and affective disorder. Schizophrenia was found to be associated with a higher risk of FI. The evidence was insufficient to support a causal relationship between Fl and other Ml. These findings offer new insights into the development of effective management strategies for frailty and MI.

## 1 Introduction

Frailty, recognized as a prevalent geriatric syndrome, encompasses diminished physiological reserve and dysregulation across multiple systems leading to an impaired capacity to sustain a stable internal environment amidst internal and external stressors. Consequently, this condition amplifies vulnerability to adverse events (1). The frailty index (FI) is acknowledged as a sensitive and effective tool for detecting frailty (2). It is a continuous metric that quantifies frailty based on the proportion of health deficits due to aging to all the number of deficits considered. These deficits may present as symptoms, signs, diseases, disabilities, or abnormalities identified through laboratory tests, radiological imaging, and even social factors (3). By encompassing various dimensions of health, the FI serves as a robust predictor of adverse outcomes, including functional decline, physical disability, falls, and increased risk of mortality and morbidity (4). Extensive epidemiological surveys have consistently demonstrated that the prevalence of frailty is increasing globally as populations age. According to a comprehensive meta-analysis, 26.8% of the older population suffers from frailty (5), and it poses a significant public health burden due to its strong association with various adverse health outcomes, such as multimorbidity, disability, and excess mortality (1). Mental illness (MI) is a primary cause of disability worldwide, and is associated with increased all-cause mortality (6). A recent meta-analysis revealed that approximately 14.3% of global deaths, which amounts to around 8 million deaths each year, are attributed to MI (7). The common conditions include anxiety, depression, affective disorder, mania, schizophrenia, and obsessive-compulsive disorder (OCD). Indeed, Prior observational studies have suggested that frailty and MI frequently coexist as overlapping syndromes in later life (8). For instance, A study involving 297,380 participants revealed identified that the likelihood of experiencing frailty among individuals with non-MI was a mere 1.8%, whereas individuals with MI exhibited frailty traits at a rate of 4.2%-5.5% (9). Furthermore, a prospective cohort study conducted in the Netherlands involving 167,729 individuals yielded similar conclusions. This study found that frailty is associated with MI, including affective disorders, anxiety, and depression (10). However, the causal link between frailty and MI remains unclear, as existing evidence from observational studies does not rule out reverse causation and confounding effects (11). Consequently, it is challenging to ascertain whether frailty leads to MI, MI contributes to frailty, or if a bidirectional causality exists. Clarifying this relationship is essential for understanding the intricate connection between frailty and MI and for devising tailored management strategies for older adults.

Randomized controlled trials (RCTs) are the gold standard for investigating causality. However, RCTs demand substantial financial and human resources, making it particularly challenging to ascertain the causal relationship between frailty and MI through such studies. In situations where conducting randomized controlled studies is not feasible, Mendelian randomization (MR) serves as an alternative technique. It utilizes genetic variation as an instrumental variable (IV) to evaluate the consistency of observational associations between risk factors and outcomes with causal effects, making it a valuable approach that provide a high level of evidence (12). Recently, the MR approaches have been successfully applied to reveal the causal role of frailty in various health outcomes (13–15). At present, there is limited research exploring the association between frailty and MI, and the existence of a causal relationship between the two remains uncertain. Therefore, this study aimed to conduct a two-sample MR analysis using published data on frailty and MI derived from genomewide association studies (GWAS). Initially, we identified single nucleotide polymorphisms (SNPs) associated with the FI, the most commonly utilized tool for frailty assessment, based on extensive GWAS. This was done to explore the causal relationship between genetic susceptibility to frailty and MI, including conditions such as anxiety, depression, affective disorders, mania, schizophrenia, and OCD. Subsequently, we carried out reverse MR analyses to assess the potential impact of MI on frailty.

## 2 Methods

### 2.1 Study design

**Figure 1:**
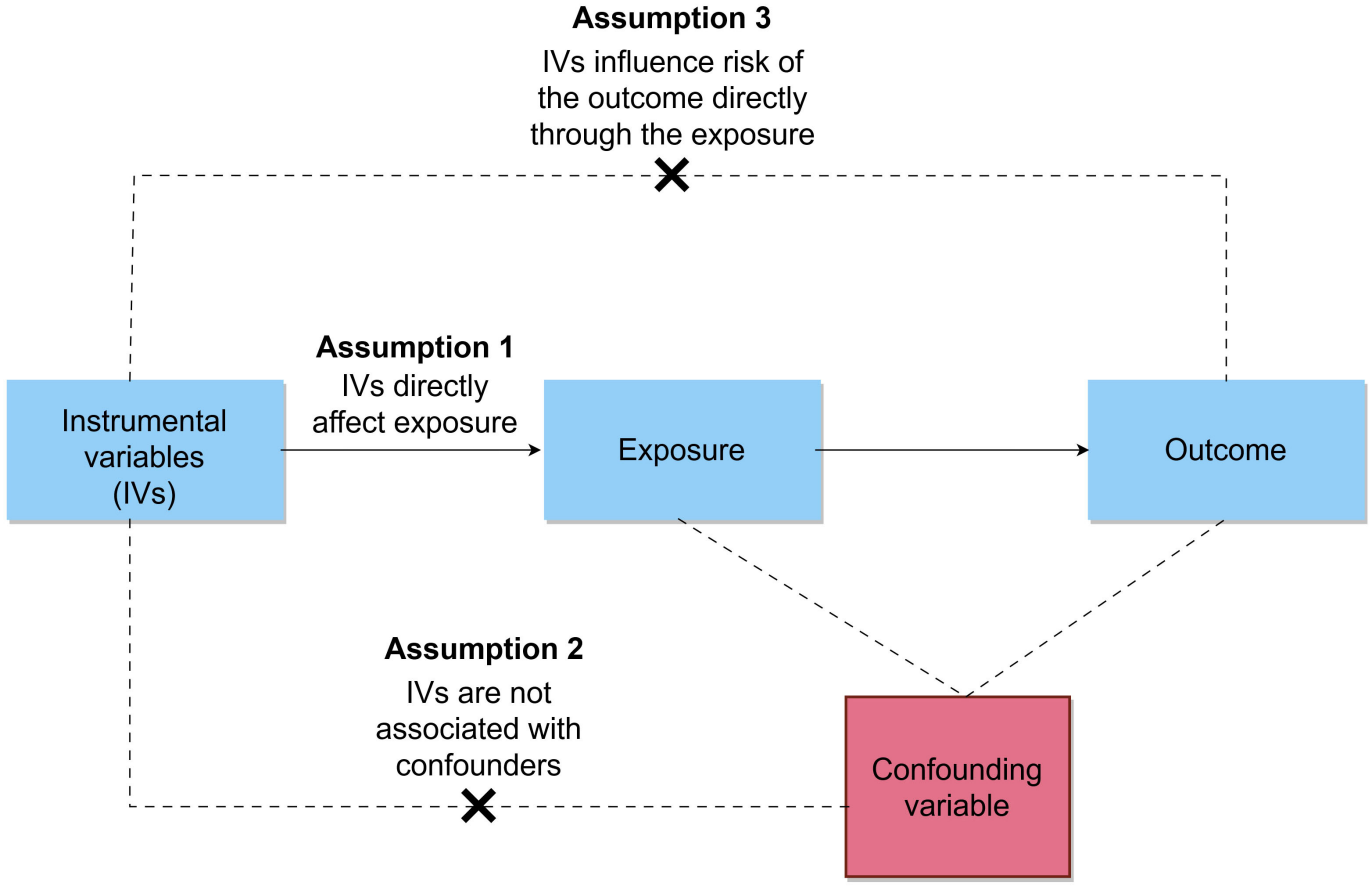
Overview of the bidirectional MR study design.

An overview of the MR framework was illustrated in **Figure 1**. This study employed a two-sample MR design that extracted summarized genetic association data for the exposure and outcome variables from two independent non-overlapping populations. The study specifically focused on individuals of European ancestry to minimize bias resulting from population stratification. To ensure the validity of causal inferences drawn from MR analysis, the IVs must satisfy three core assumptions: (i) the relevance assumption, meaning the SNPs are strongly associated with the exposure; (ii) the independence assumption, indicating the SNPs should not be associated with confounding factors; (iii) the exclusion-restriction assumption, suggesting that SNPs affect the outcome solely through the exposure.

MR analysis is conducted from two perspectives: (i) frailty as the exposure, assessing whether individuals with a higher FI are more likely to develop MI; (ii) frailty as the outcome, evaluating whether patients with MI are more likely to be frailty.

### 2.2 Data source and genetic instrument selection

Detailed information is shown in **Table 1**. Summary statistics for frailty, as assessed by the FI phenotype, were acquired from a comprehensive meta-analysis of GWAS conducted within the UK Biobank and Swedish TwinGene cohorts (16). These datasets encompassed a substantial sample size of 175,226 individuals of European ancestry, enabling a robust exploration of the genetic underpinnings of frailty. FI was calculated based on 49 or 44 self-reported items according to UK Biobank and TwinGene’s defect accumulation theory, respectively (16, 17). We selected publicly available summary statistic data sets of a GWAS for anxiety (total N = 395,718; case = 27,554, control = 368,054), depression (total N = 406,986; case = 47,696, control = 359,290), affective disorder (total N = 412,181; case = 52,891, control = 359,290) in FinnGen R10 dataset. Mania data from UK Biobank (total N = 115,338; case = 4,816, control = 110,522). Schizophrenia data from a recent large GWAS study (total N = 320,404; case = 76,755, control = 243,649) (18). In addition, we obtained GWAS data for OCD from the Psychiatric Genomics Consortium (total N = 33,925; case = 26,888, control = 7,037) (19).

**Table 1:** Summary of GWAS included in this study.

Year	Trait	Population	Cases	Controls	Samplesize	Websource
2021	Frailty	European	NA	NA	175,226	DOI: 10.1111/acel.13459
2023	Anxiety	European	27,554	368,054	395,718	www.finngen.fi/en
2023	Depression	European	47,696	359,290	406,986	www.finngen.fi/en
2023	Affective disorder	European	52,891	359,290	412,181	www.finngen.fi/en
2018	Mania	European	4,816	110,522	115,338	www.nealelab.is/uk-biobank
2022	Schizophrenia	European	76,755	243,649	320,404	https://pgc.unc.edu/
2017	Obsessive-compulsive disorder	European	26,888	7,037	33,925	https://pgc.unc.edu/

The selection of IVs adhered strictly to the three fundamental assumptions of MR analysis. Initially, SNPs significantly associated with the exposure were identified based on genome-wide significance (*P* < 5E-8) and subsequently grouped to ensure SNP independence (cluster r^2^ cutoff = 0.001, cluster distance = 10,000 kb). In cases of linkage disequilibrium (LD) among SNPs, the SNP with the lowest p-value was retained. Palindromic SNPs, SNPs associated with the outcome (*P* < 0.05), and SNPs absent from the GWAS pooled data were excluded from the IV selection. Given that a portion of the GWAS data in FI and mania in this study was derived from the UK bioBank, which may lead to potential data overlap, we took steps to mitigate the impact of such overlap on the MR analysis. Specifically, only SNPs with F-statistics greater than 10 were included in subsequent analysis (20, 21). Additionally, PhenoScanner V2, an advanced tool for identifying human genotype-phenotype associations, was utilized to check if the selected SNPs were linked to confounders in the frailty and MI relationship (threshold: 1E-05). Confounders identified were adjusted for in subsequent analyses.

### 2.3 Statistical analysis

Before conducting the MR analysis, we initially employed MR Pleiotropy RESidual Sum and Outlier (MR-PRESSO) to identify and discard any abnormal IVs. The MR-PRESSO procedure was performed with a cycle number of 10,000 and *P* < 0.05 was used as a threshold to detect and remove outliers. Considering the significant heterogeneity of SNP effects, we applied the multiplicative random-effects model in the inverse variance weighting (IVW) approach as the main analytical method for MR, which is an extension of the Wald ratio estimator based on the principles of meta-analysis (22). The significance threshold was set at *P <*0.05 and the results of causality were expressed as odds ratios (OR) and 95% confidence intervals (95% CI). To further demonstrate the stability and directionality of the results, we additionally performed MR-Egger and weighted median to assess causality. These methods rely on different assumptions, so the consistent effects of multiple methods can lead to causal conclusions with greater persuasiveness (23). Next, to test the robustness of our results, we assessed heterogeneity using Cochran’s Q (24). Then, horizontal pleiotropy was tested using the MR-Egger intercept and MR-PRESSO global test (25). Finally, we also performed sensitivity analyses through funnel plots and leave-one-out methods.

We further performed reverse MR analysis to assess whether anxiety or depression affects frailty. Due to the limited number of SNPs meeting the genome-wide significance threshold (*P* < 5E-8) in the GWAS of the pooled dataset for part MI, we conducted a screening for SNPs meeting a more relaxed genome-wide significance threshold (*P* < 5E-6) to serve as IVs associated with MI (26). The subsequent method is the same.

All analyses in this study were performed based on R software (version 4.2.1). The “TwoSampleMR” R package was used in our MR study (27). All statistical tests were two tailed, and α = 0.05 was considered as the significant level.

## 3 Results

### 3.1 Instrumental variables for Mendelian randomization

This study investigates the impact of FI on MI risk using two-sample MR. A set of 15 SNPs, associated with FI and independent from other factors, were selected as IVs from the GWAS dataset. When employing PhenoScanner, IVs linked to pertinent potential confounders were identified by applying a threshold of 1E-05. These confounders were subsequently omitted from the formal MR analyses, resulting in the exclusion of 5 SNPs, the specifics of which are delineated in **Supplementary Table 1**. The remaining 10 SNPs as IVs associated with frailty are shown in **Supplementary Table 2**.

Examined a set of SNPs associated with anxiety, depression, affective disorders, mania, schizophrenia, and OCD. Specifically, we screened 18, 17, 21, 19, 217, and 15 significant and independent SNPs from each respective disorder to serve as IVs. PhenoScanner was used to eliminate 6, 6, 6, 6, 57, and 3 SNPs associated with confounders, the specifics of which are delineated in **Supplementary Table 1**. The remaining 12,11, 14, 17, 160 and 12 SNPs as IVs associated with MI are shown in **Supplementary Tables 3**-**8**.

### 3.2 The effect of frailty index on the risk of mental illness

**Figure 2:**
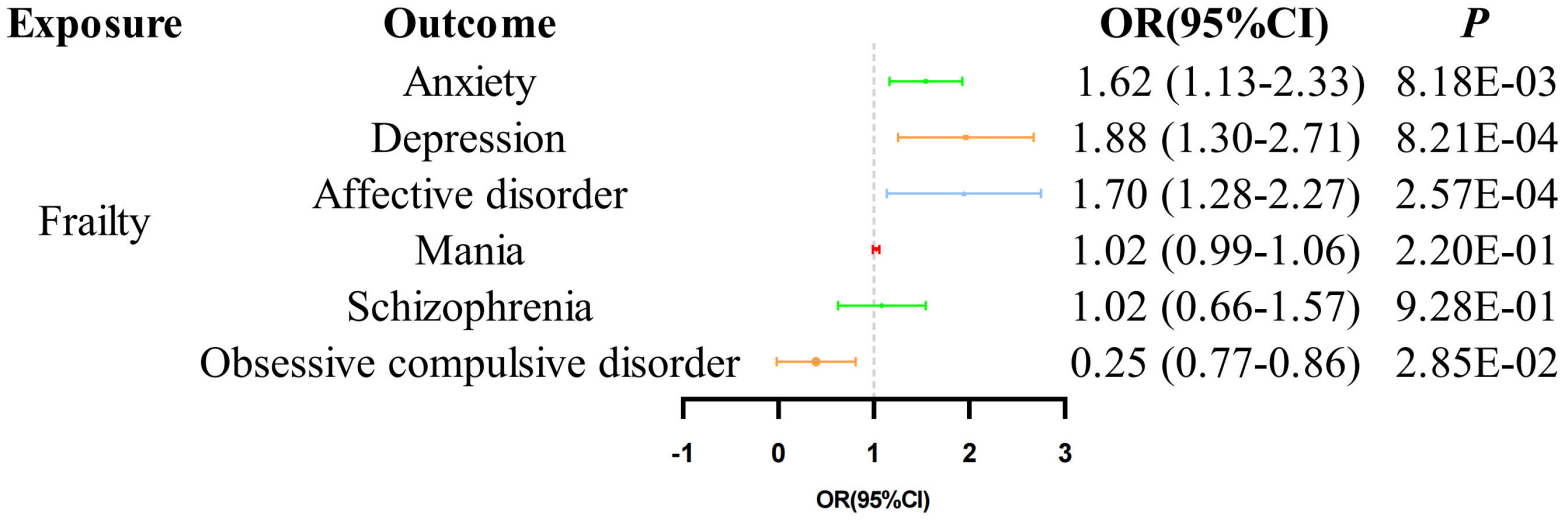
MR analysis of the causal correlation between FI on the risk of MI.

The results of the MR analysis indicate a causal relationship between FI and multi-MI. The MR results using the IVW method revealed that FI was associated with a significantly increased risk of anxiety (OR = 1.62, 95% CI 1.13-2.33, *P* = 8.18E-03), depression (OR = 1.88, 95% CI 1.30-2.71, *P* = 8.21E-04) and affective disorder (OR = 1.70, 95% CI 1.28-2.27, *P* = 2.57E-04). However, our results do not support a causal relationship between FI on the risk of mania (OR = 1.02, 95% CI 0.99-1.06, *P* = 2.20E-01) and schizophrenia (OR = 1.02, 95% CI 0.66-1.57, *P* = 9.28E-01). In particular, the results of the IVW analysis indicated a negative correlation between FI and the risk of OCD (OR = 0.25, 95% CI 0.07-0.86, *P* = 2.85E-02). However, it is important to interpret these results with caution, as the three methods used in this study were not consistent in their direction.The detail results of the MR analysis are shown in **Table 2**, **Figure 2**. Further, the Scatter plots and forest plots of the SNP-outcome associations against the SNP-exposure associations are displayed in **Supplementary Figures 1**, **2**. Cochran’s Q statistic results revealed significant heterogeneity when examining the causal effect of FI on depression (*P* = 0.03) and schizophrenia (*P* = 0.01), whereas no significant heterogeneity was observed in the effect of SNPs across the remaining studies. The MR-Egger regression analysis did not find any evidence of horizontal pleiotropy, and no significant outlier was further identified by MRPRESSO. The results of “leave one out” indicate that there is no single SNP that has a large role in driving the outcome (**Supplementary Figures 3**). Additionally, the funnel plot provides further evidence that the study is unbiased (**Supplementary Figure 4**). The results of the sensitivity analysis of the MR analysis are shown in **Table 3**.

**Table 2:** MR estimates from each method of assessing the causal effect of frailty on the risk of psychiatric illness.

Exposure	Outcome	MR method	OR	Beta	SE	95% confidence interval	*P* value
Frailty	Anxiety	IVW	1.62	0.48	0.18	1.13-2.33	8.18E-03
		MR Egger	2.13	0.76	0.77	0.47-9.65	3.58E-01
		Weighted median	1.37	0.46	0.20	1.07-2.33	2.19E-02
	Depression	IVW	1.88	0.63	0.19	1.30-2.71	8.21E-04
		MR Egger	3.25	1.18	0.72	0.79-13.31	1.62E-01
		Weighted median	2.00	0.69	0.18	1.40-2.84	1.25E-04
	Affective disorder	IVW	1.70	0.53	0.15	1.28-2.27	2.57E-04
		MR Egger	3.57	1.27	0.47	1.43-8.94	7.24E-02
		Weighted median	1.88	0.63	0.17	1.34-2.64	2.37E-04
	Mania	IVW	1.02	0.02	0.02	0.99-1.06	2.20E-01
		MR Egger	1.13	0.13	0.06	1.01-1.27	6.93E-02
		Weighted median	1.03	0.03	0.02	1.00-1.07	4.23E-02
	Schizophrenia	IVW	1.02	0.02	0.22	0.66-1.57	9.28E-01
		MR Egger	15.44	2.74	1.70	0.56-429.13	1.51E-01
		Weighted median	1.18	0.17	0.22	0.77-1.81	4.38E-01
	Obsessive-compulsive disorder	IVW	0.25	-1.40	0.64	0.07-0.86	2.85E-02
		MR Egger	0.94	1.60	5.87	5.02E-05-4.86E+05	7.94E-01
		Weighted median	0.24	-1.42	0.82	0.05-1.20	8.17E-02

**Table 3:** Sensitivity analysis of the MR analysis results of exposures and outcomes.

Exposure	Outcome	Heterogeneity test	Pleiotropy test	MR-PRESSO*	
		Cochran’s Q test (*P* value) IVW	Egger intercept (*P* value) MR-egger	Distortion test Outliers	Global test *P* Value
Frailty	Anxiety	0.11	0.73	NA	0.15
	Depression	0.03	0.46	NA	0.07
	Affective disorder	0.29	0.20	NA	0.37
	Mania	0.07	0.11	NA	0.10
	Schizophrenia	0.01	0.15	NA	0.02
	Obsessive-compulsive disorder	0.68	0.63	NA	0.69
Anxiety	Frailty	0.33	0.61	NA	0.39
Depression		0.05	0.86	NA	0.08
Affective disorder		0.31	0.77	NA	0.36
Mania		0.05	0.71	NA	0.05
Schizophrenia		<0.001	0.93	NA	<0.001
Obsessive-compulsive disorder		0.13	0.48	NA	0.12

*Results of MR-PRESSO global test are presented here. For MR analyses with a global test, *P < 0.05*, no significant outlier was detected by MR-PRESSO.

### 3.3 Results of reverse Mendelian randomization analysis

**Figure 3:**
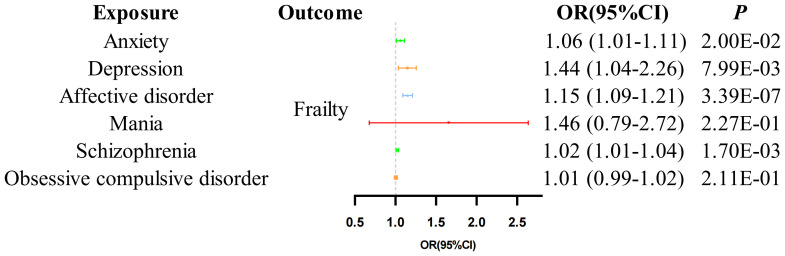
MR analysis of the causal correlation between MI on the risk of FI.

The investigation into the causal relationship between multi-MI and the risk of FI reveals compelling evidence through MR analysis. According to the primary IVW, genetically predicted anxiety (OR = 1.06, 95% CI 1.01-1.11, *P* = 2.00E-02), depression (OR = 1.14, 95% CI 1.04-1.26, *P* = 7.99E-03), affective disorder (OR = 1.15, 95% CI 1.09-1.21, *P* = 3.39E-07), and schizophrenia (OR = 1.02, 95% CI 1.01-1.04, *P* = 1.70E-03) were identified as risk factors for FI. However, according to the IVW results, there was no evidence of a causal relationship between mania (OR = 1.46, 95% CI 0.79-2.72, *P* = 2.27E-01) and OCD (OR = 1.01, 95% CI 0.99-1.02, *P* = 2.11E-01) with FI. The detail results of the MR analysis are shown in **Table 4**, **Figure 3**. Further, the scatter plots and forest plots of the SNP-outcome associations against the SNP-exposure associations are displayed in **Supplementary Figures 5**, **6**. The results of Cochran’s Q statistic results revealed significant heterogeneity when examining the causal effect of schizophrenia (*P* < 0.001) on FI, whereas no significant heterogeneity was observed in the effect of SNPs across the remaining studies. The MR-Egger regression analyses indicated that the presence of multiplicity affecting the results was unlikely, and no significant outlier was further identified by MRPRESSO. Additionally, we visually examined sensitivity using “leave one out” (**Supplementary Figure 7**) and funnel plots (**Supplementary Figure 8**), which confirmed the robustness of our results. The results of the sensitivity analysis of the MR analysis are shown in **Table 3**.

**Table 4:** MR estimates from each method of assessing the causal effect of psychiatric illness on the risk of frailty.

Exposure	Outcome	MR method	OR	Beta	SE	95% confidence interval	*P* value
Anxiety	Frailty	IVW	1.06	0.06	0.02	1.01-1.11	2.00E-02
		MR Egger	1.01	0.01	0.10	0.84-1.22	9.42E-01
		Weighted median	1.05	0.06	0.03	0.99-1.12	9.46E-02
Depression		IVW	1.14	0.13	0.05	1.04-1.26	7.99E-03
		MR Egger	1.21	0.20	0.34	0.62-2.39	5.91E-01
		Weighted median	1.14	0.13	0.05	1.03-1.27	1.51E-02
Affective disorder		IVW	1.15	0.14	0.03	1.09-1.21	3.39E-07
		MR Egger	1.07	0.07	0.24	0.67-1.72	7.80E-01
		Weighted median	1.15	0.14	0.04	1.08-1.24	7.52E-05
Mania		IVW	1.46	0.38	0.32	0.79-2.72	2.27E-01
		MR Egger	1.16	0.15	0.68	0.31-4.38	6.77E-01
		Weighted median	0.88	-0.12	0.34	0.46-1.71	3.38E-01
Schizophrenia		IVW	1.02	0.01	0.02	1.01-1.04	1.70E-03
		MR Egger	1.03	0.03	0.02	0.97-1.07	4.36E-01
		Weighted median	1.02	0.01	0.02	1.00-1.04	3.30E-02
Obsessive-compulsive disorder		IVW	1.01	0.01	0.01	0.99-1.02	2.10E-01
		MR Egger	1.02	0.02	0.02	0.98-1.07	3.12E-01
		Weighted median	1.01	0.01	0.01	0.99-1.02	4.00E-01

## 4 Discussion

Using several large-scale GWAS data, we performed a bidirectional MR analysis to estimate the causal relationship between frailty and MI. We observed bidirectional causality between FI and anxiety, depression, and affective disorders. FI had no significant effect on the risk of mania, schizophrenia and OCD. In contrast, schizophrenia was associated with higher FI, and there was no reliable evidence to support genetically predicted effects of manic and OCD on FI. Although several previous MR studies have explored the causal relationship between frailty and some MI (17, 28–31), our study possesses several strengths worth noting. First, this study is the first to explore the causal relationship between FI and affective disorders, mania, and OCD through MR. Secondly, our data were sourced from the latest version of the database and enhancing the reliability of our results and updating for previous findings. This finding offers valuable insights into the association between FI and MI, laying a theoretical groundwork for future development of public health policies.

Previous observational studies have indicated a possible link between the vulnerability index, a clinical metric of biological age in the field of psychiatry, and MI (32). Both individuals classified as frail and pre-frail exhibited significantly diminished scores in mental and physical quality of life compared to non-frail individuals (33). A 25-year study utilizing replicated data revealed that individuals experiencing frailty exhibited significantly lower scores in the Short-Form 36 General Health Survey (SF-36) in comparison to non-frailty individuals (34). For instance, a prior study conducted in western China, involving 4,103 community residents aged 60 years and older, revealed that individuals grappling with co-morbidities of depression and anxiety exhibited elevated odds of pre-frailty (OR=1.86, 95% CI=1.41-2.45) and frailty (OR=7.03, 95% CI=4.48-11.05) in contrast to individuals without depressive and anxiety symptoms (35). In addition, Recent studies have shown a correlation between affective disorders and frailty, as well as an increased risk of relapse in frail patients (10, 36). Furthermore, the COVID-19 pandemic appears to have resulted in a heightened severity of frailty and MI. Studies have demonstrated that pre-existing frailty in older adults was correlated with increased likelihood of enduring and abrupt MI during the initial wave of the COVID-19 pandemic, and this association persisted (37, 38). Notably, several studies have indicated a high prevalence of frailty among individuals diagnosed with schizophrenia, including those in younger age groups (39, 40). Recent studies also have demonstrated strikingly similar patterns of gene activity in aging and schizophrenic patients, particularly in neurons and astrocytes in the prefrontal cortex (41). This indicates a potential association between frailty and schizophrenia. Similarly, our findings indicate a link between FI and schizophrenia, suggesting that a state of schizophrenia may elevate the risk of frailty, though the evidence for reverse causality is weak. However, it has to be recognized that when examining the impact of schizophrenia on the risk of frailty onset, the OR was only 1.02 with a confidence interval of 1.01-1.04, which was also confirmed by the Weighted median approach. This suggests that schizophrenia may not significantly heighten the risk of frailty. Theoretically, MR evaluates the lifelong impact of genetically predicted exposures on the incidence of an outcome over an extended duration, often yielding results with more pronounced effect sizes compared to traditional observational studies (42, 43). This aspect should be considered when interpreting our findings, leading us to adopt a conservative stance in observing a modest association between frailty and schizophrenia. Future observational studies examining the relationship between frailty and schizophrenia should be conducted in larger populations, and a more extensive population-based GWAS for mania is warranted. Previous observational studies have given little attention to OCD in older adults. Some studies suggest that individuals with OCD experience accelerated brain aging, as well as shortening of mitochondrial DNA copy number (mtDNAcn) and telomere length in the blood (44, 45). However, our study did not observe a bidirectional relationship between FI and OCD. We consider the lack of association found in our analysis to be relatively reliable. Observational studies can be confounded by confounders and reverse causation, making it difficult to determine a causal relationship between variables. Our study utilized the MR method, which is less susceptible to confounding bias than traditional observational designs (46). To confirm our findings, Further studies based on GWAS data with larger sample sizes and representative participants are needed.

The exact mechanisms underlying the relationship between frailty and MI have yet to be fully understood. Various hypotheses can explain the bidirectional relationship between frailty and MI. First, physical weakness can result in diminished physical activity and social interaction, as well as increased sedentary behavior, ultimately contributing to the onset of MI (47, 48). Conversely, MI can give rise to unfavorable symptoms, such as reduced social interaction, weight loss, and malnutrition (49, 50). Secondary, frailty and MI share numerous risk factors, including chronic inflammation (51, 52), cardiovascular disease (53, 54), and unhealthy lifestyle choices (23, 55). Thirdly, treatments for frailty or MI that had beneficial effects can also protect each other. For instance, it was well-known that incorporating physical activity as a strategy can enhance physical functioning in older and frail individuals while also benefiting reasoning and problem-solving abilities in individuals with MI, improving their symptoms in the process (56–58). Therefore, the presence of a bidirectional relationship between frailty and MI is not coincidental, as all the evidence substantiates this hypothesis.

The escalating global burden of frailty underscores the urgent need to slow down its progression and enhance the well-being and quality of life of older adults (59). A recent study has revealed a significant co-occurrence of frailty and mental disorders, leading to heightened mortality rates (9). This study’s results comprehensively evaluated the bidirectional causal relationship between FI and MI, minimizing potential confounding biases. These reciprocal findings on FI and MI carry critical implications for public health and clinical practice. Primarily, there is a crucial necessity to fortify efforts in identifying and managing frailty, implementing timely interventions in its early stages. Additionally, our results underscore the importance of psychologically relevant strategies, including routine screening for psychological disorders, social support, and targeted psychological interventions, essential for both primary and secondary prevention of frailty to avert unfavorable outcomes and break vicious cycles. From a public health perspective, our findings could inform the development of management strategies addressing common risk factors and interventions to prevent frailty and MI in older adults, thereby alleviating adverse outcomes and enhancing their overall quality of life.

This study is subject to certain limitations. Firstly, it exclusively considers individuals of European descent, thus failing to address potential genetic variations across different races, countries, and regions. Secondly, the reliance on pooled data limits the availability of detailed clinical information, which restricts our ability to perform analyses to gender specificity. Thirdly, the limited number of SNPs meeting the genome-wide significance threshold for the GWAS of the pooled dataset for MI may have impacted the reverse MR analysis. Fourthly, It is crucial to acknowledge that MR analyses inherently offer less robust evidence of causality compared to RCTs. Fifthly, part of the FI data and mania’s summarized GWAS data came from the same database, which led to an overlap in the sample populations between the studies, as some participants were included in both studies. To address these limitations, future large-scale GWAS studies should be conducted across diverse ethnicities and differentiate between their sexes. In addition, there is a pressing need for additional high-quality RCTs to corroborate and fortify these findings. However, it is ethically challenging to explore the causal relationship between FI and MI through RCTs; therefore, extended prospective cohort studies may serve as a viable alternative. Furthermore, exploring the efficacy of interventions tailored to target common risk factors for FI and MI in enhancing patient care management warrants further investigation in forthcoming trials.

## 5 Conclusion

In summary, this study identified a reciprocal association between frailty and risk of MI using MR methods. On the basis of our findings, it is reasonable to consider promoting routine frailty screening in MI patients. In addition, proper management of MI is also essential for downregulating the risk of frailty.

## Data availability statement

The original contributions presented in the study are included in the article/**Supplementary Material**. Further inquiries can be directed to the corresponding authors.

## Ethics statement

All data analyzed in this study were obtained from publicly available databases in which ethical approval was obtained for each cohort, and informed consent was obtained from all participants prior to participation.

## Author contributions

LM: Data curation, Formal analysis, Writing – original draft. ZL: Data curation, Writing – original draft. LF: Formal analysis, Writing – original draft. JF: Formal analysis, Writing – original draft, Writing – review & editing. CK: Writing – original draft. TW: Writing – original draft. HB: Writing – review & editing. QL: Writing – review & editing. JY: Conceptualization, Writing – review & editing. XF: Conceptualization, Project administration, Writing – review & editing.

## Supplementary material

Supplementary Figure 1 Scatter plots of single SNP effect and estimates from multiple two-sample MR analyses for the causal effect of FI on anxiety **(A)**, depression **(B)**, affective disorder **(C)**, mania **(D)**, schizophrenia **(E)**, and OCD **(F)** in replication analysis.

Supplementary Figure 2 Forest plots of causal effects of FI on anxiety **(A)**, depression **(B)**, affective disorder **(C)**, mania **(D)**, schizophrenia **(E)**, and OCD **(F)**. The bars indicate the confidence interval of MR estimates.

Supplementary Figure 3 Leave-one-out plots of two-sample Mendelian randomization analysis for genetically predicted FI on anxiety **(A)**, depression **(B)**, affective disorder **(C)**, mania **(D)**, schizophrenia **(E)**, and OCD **(F)** outcomes. The dots indicate MR estimates for using inverse-variance weighted method when the SNP was removed. The bars indicate the confidence interval of MR estimates.

Supplementary Figure 4 Funnel plots assess the presence of potential heterogeneity across genetic instruments for FI on anxiety **(A)**, depression **(B)**, affective disorder **(C)**, mania **(D)**, schizophrenia **(E)**, and OCD **(F)**, which indicates possible pleiotropic effects. The causal effect of each genetic instrument was presented by dots, and combined causal effect by inverse variance weighted and MR Egger were depicted by lines.

Supplementary Figure 5 Scatter plots of single SNP effect and estimates from multiple two-sample MR analyses for the causal effect of anxiety **(A)**, depression **(B)**, affective disorder **(C)**, mania **(D)**, schizophrenia **(E)**, and OCD **(F)** on FI in replication analysis.

Supplementary Figure 6 Forest plots of causal effects of anxiety **(A)**, depression **(B)**, affective disorder **(C)**, mania **(D)**, schizophrenia **(E)**, and OCD **(F)** on FI. The bars indicate the confidence interval of MR estimates.

Supplementary Figure 7 Leave-one-out plots of two-sample Mendelian randomization analysis for genetically predicted anxiety **(A)**, depression **(B)**, affective disorder **(C)**, mania **(D)**, schizophrenia **(E)**, and OCD **(F)** on FI outcomes. The dots indicate MR estimates for using inverse-variance weighted method when the SNP was removed. The bars indicate the confidence interval of MR estimates.

Supplementary Figure 8 Funnel plots assess the presence of potential heterogeneity across genetic instruments for anxiety **(A)**, depression **(B)**, affective disorder **(C)**, mania **(D)**, schizophrenia **(E)**, and OCD **(F)** on FI, which indicates possible pleiotropic effects. The causal effect of each genetic instrument was presented by dots, and combined causal effect by inverse variance weighted and MR Egger were depicted by lines.
