# Supplementary figures and images for: Bidirectional causal relational between frailty and mental illness: a two-sample Mendelian randomization study
Source: Front Psychiatry. 2024 Jun 7;15:1397813. doi: 10.3389/fpsyt.2024.1397813 (PMC11190300; doi:10.3389/fpsyt.2024.1397813)

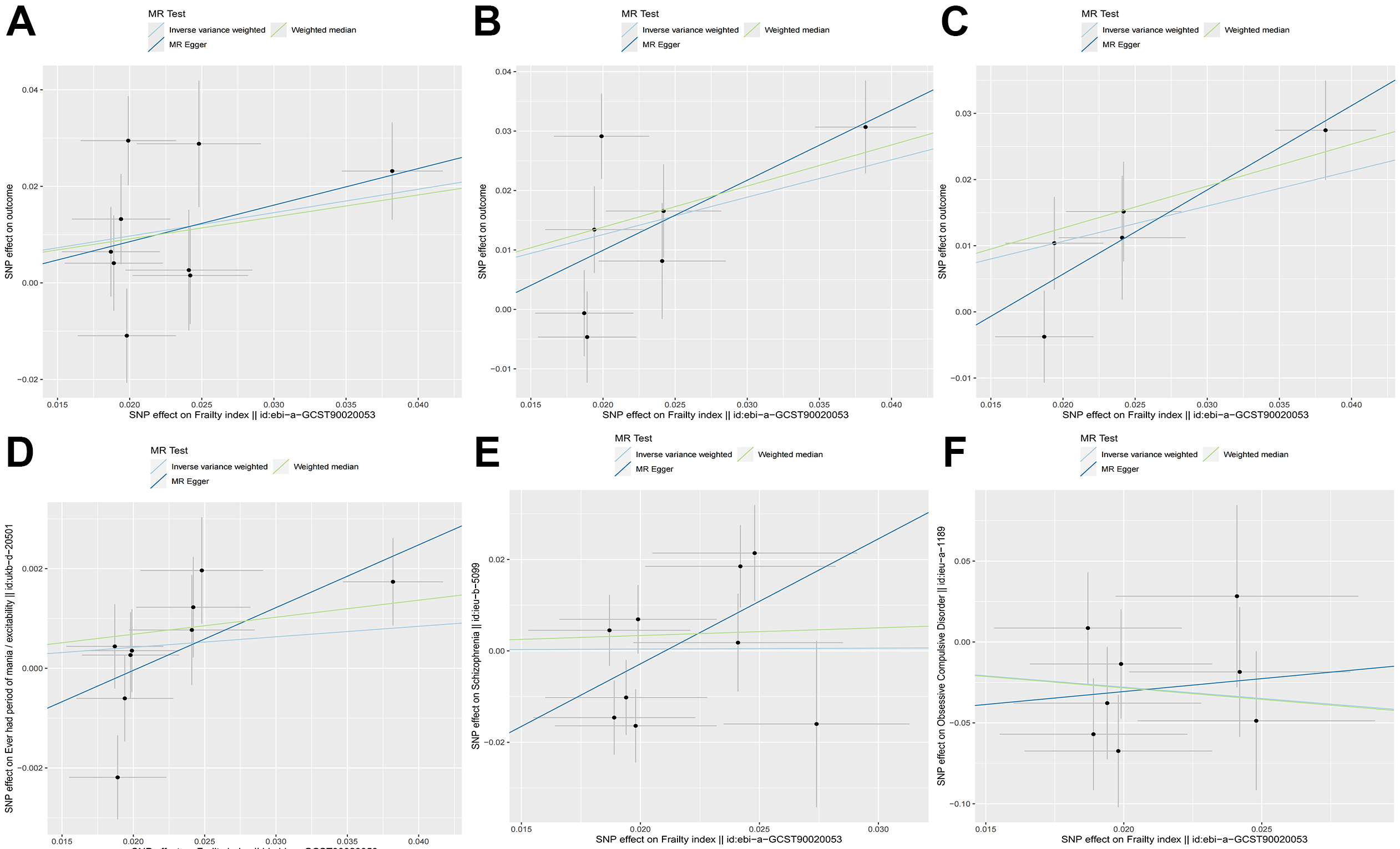

Supplement: Supplementary Figure 1 — Scatter plots of single SNP effect and estimates from multiple two-sample MR analyses for the causal effect of FI on anxiety (A), depression (B), affective disorder (C), mania (D), schizophrenia (E), and OCD (F) in replication analysis. [file Image_1.tif]

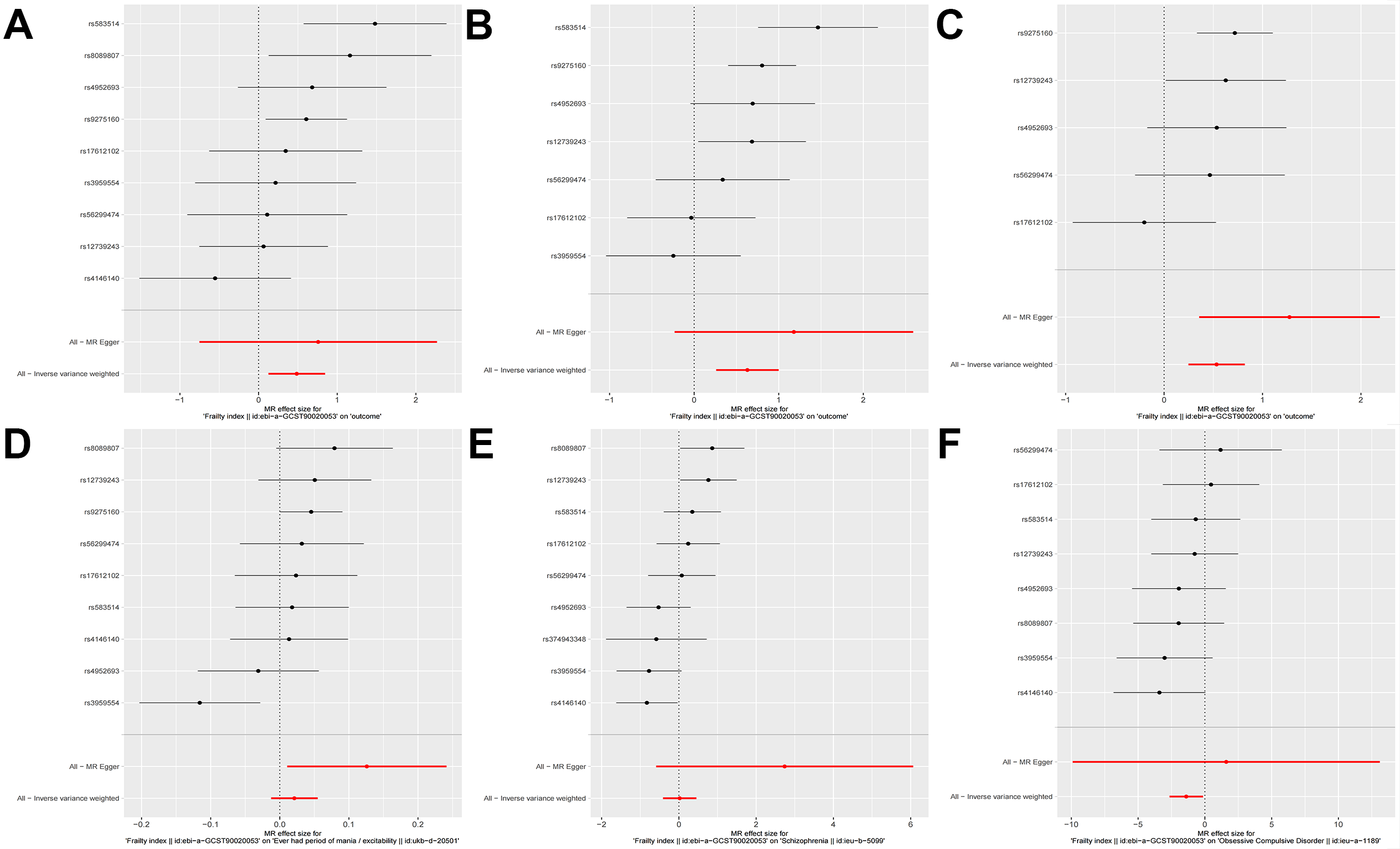

Supplement: Supplementary Figure 2 — Forest plots of causal effects of FI on anxiety (A), depression (B), affective disorder (C), mania (D), schizophrenia (E), and OCD (F). The bars indicate the confidence interval of MR estimates. [file Image_2.tif]

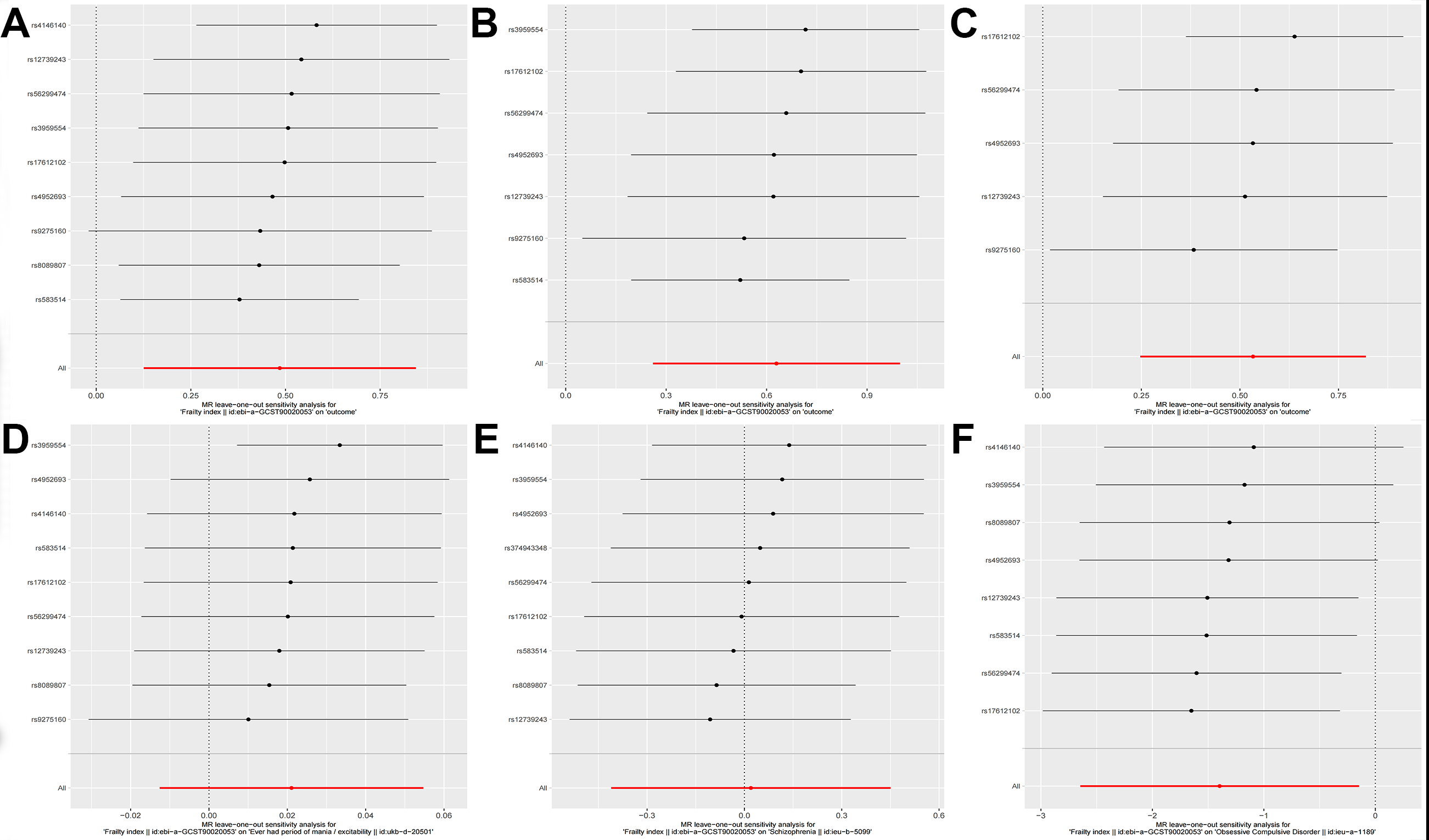

Supplement: Supplementary Figure 3 — Leave-one-out plots of two-sample Mendelian randomization analysis for genetically predicted FI on anxiety (A), depression (B), affective disorder (C), mania (D), schizophrenia (E), and OCD (F) outcomes. The dots indicate MR estimates for using inverse-variance weighted method when the SNP was removed. The bars indicate the confidence interval of MR estimates. [file Image_3.tif]

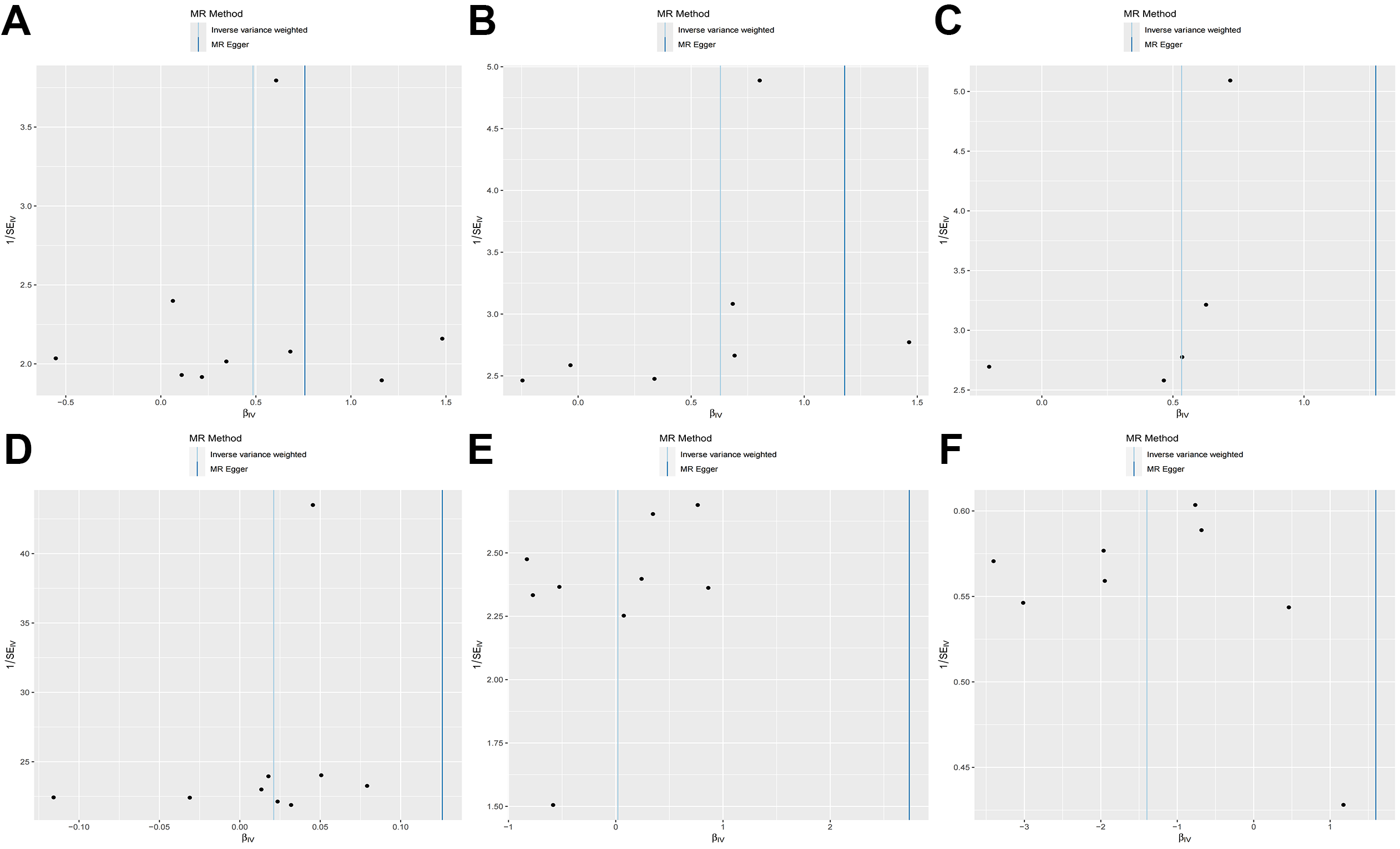

Supplement: Supplementary Figure 4 — Funnel plots assess the presence of potential heterogeneity across genetic instruments for FI on anxiety (A), depression (B), affective disorder (C), mania (D), schizophrenia (E), and OCD (F), which indicates possible pleiotropic effects. The causal effect of each genetic instrument was presented by dots, and combined causal effect by inverse variance weighted and MR Egger were depicted by lines. [file Image_4.tif]

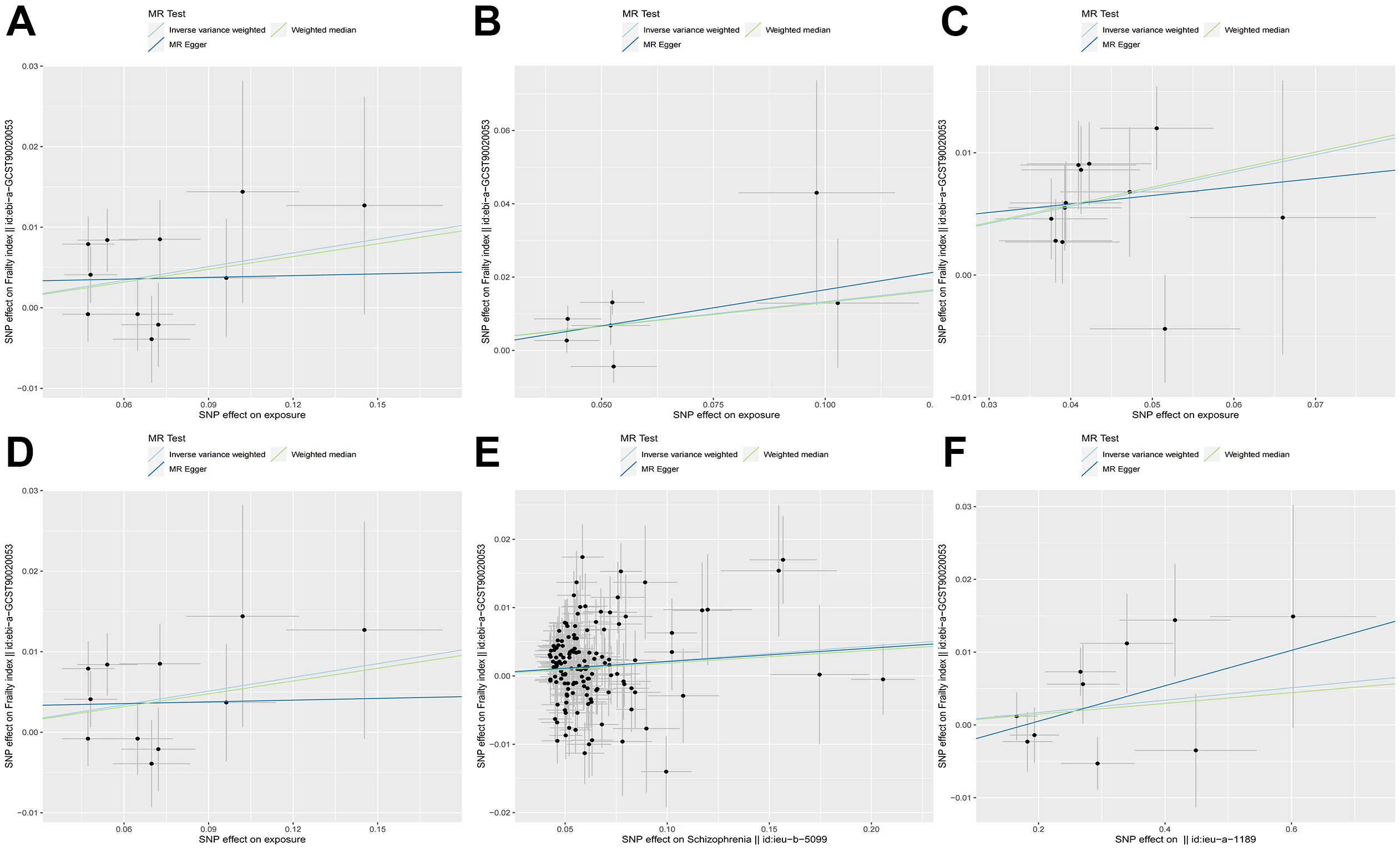

Supplement: Supplementary Figure 5 — Scatter plots of single SNP effect and estimates from multiple two-sample MR analyses for the causal effect of anxiety (A), depression (B), affective disorder (C), mania (D), schizophrenia (E), and OCD (F) on FI in replication analysis. [file Image_5.tif]

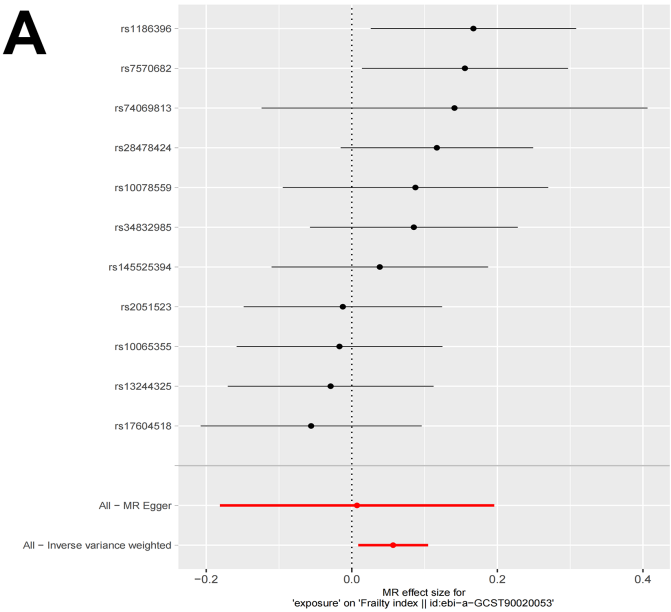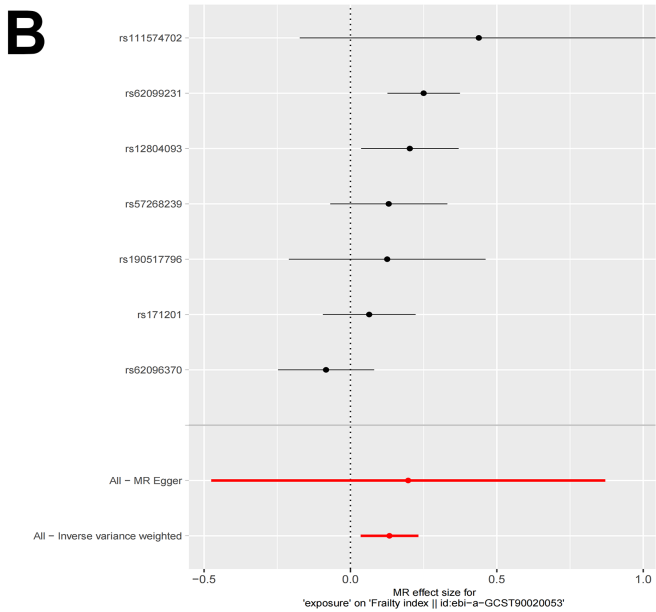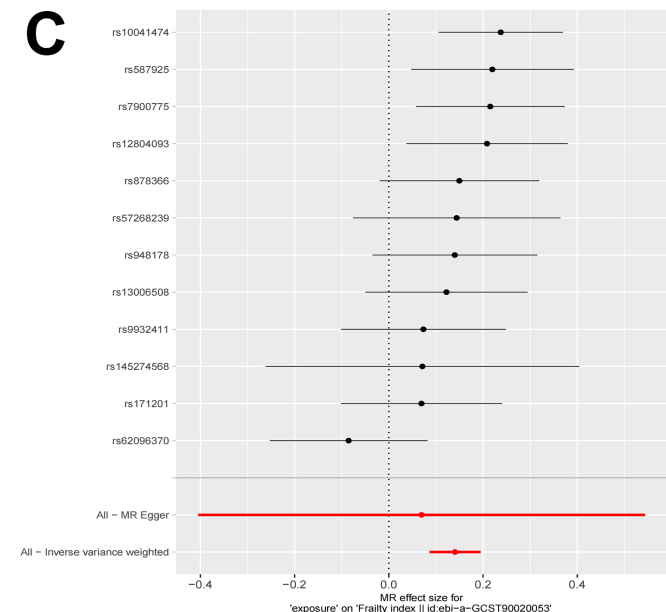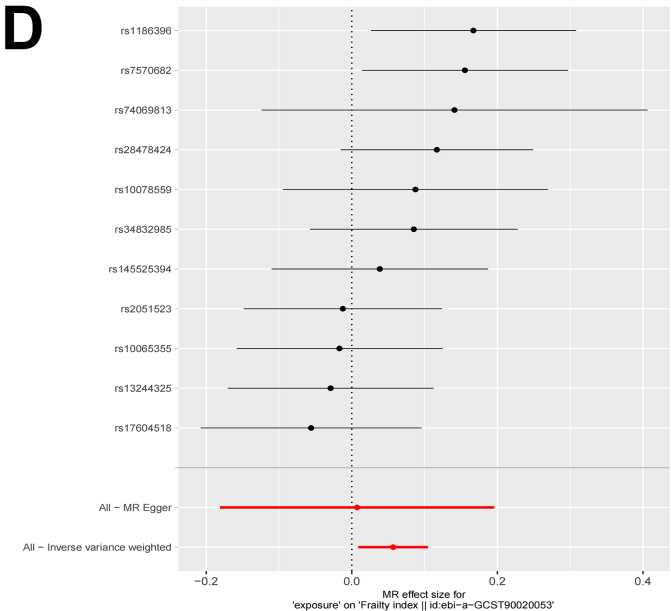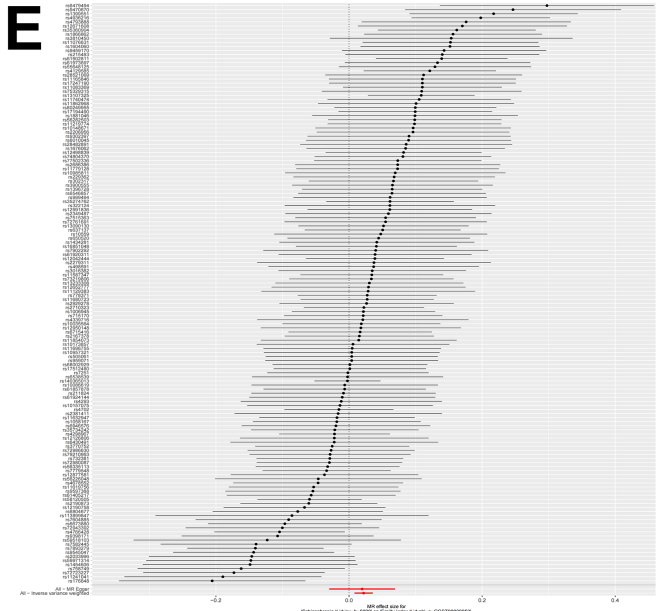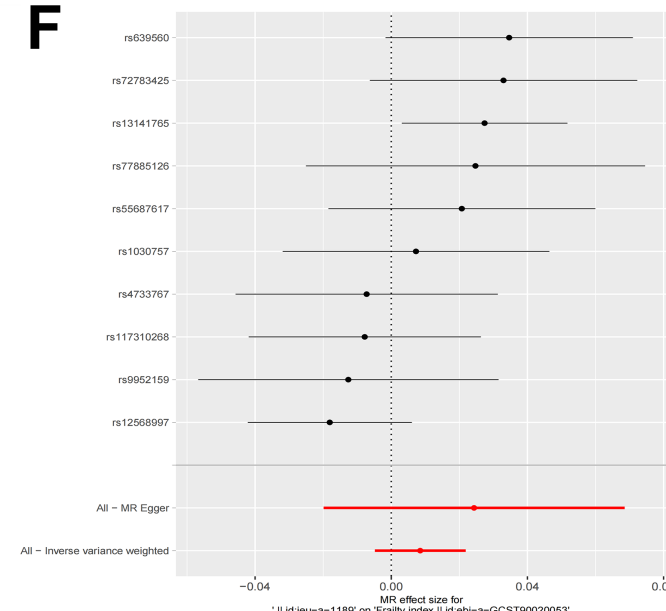

Supplement: Supplementary Figure 6 — Forest plots of causal effects of anxiety (A), depression (B), affective disorder (C), mania (D), schizophrenia (E), and OCD (F) on FI. The bars indicate the confidence interval of MR estimates. [file Image_6.pdf]

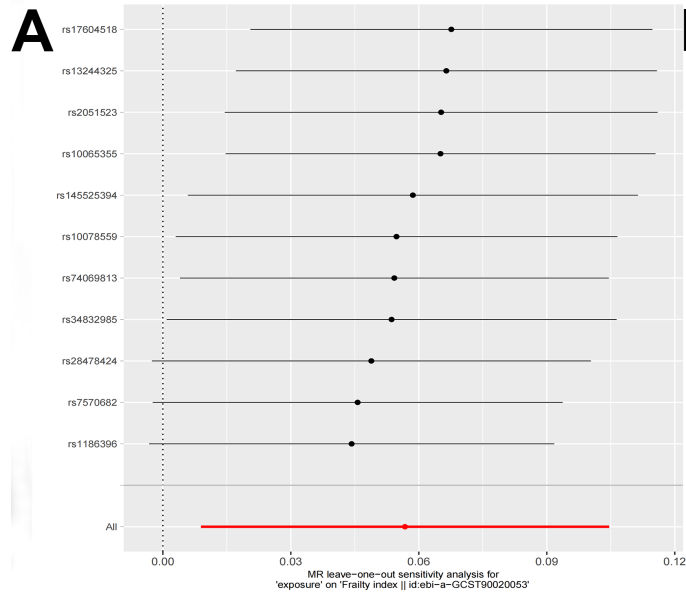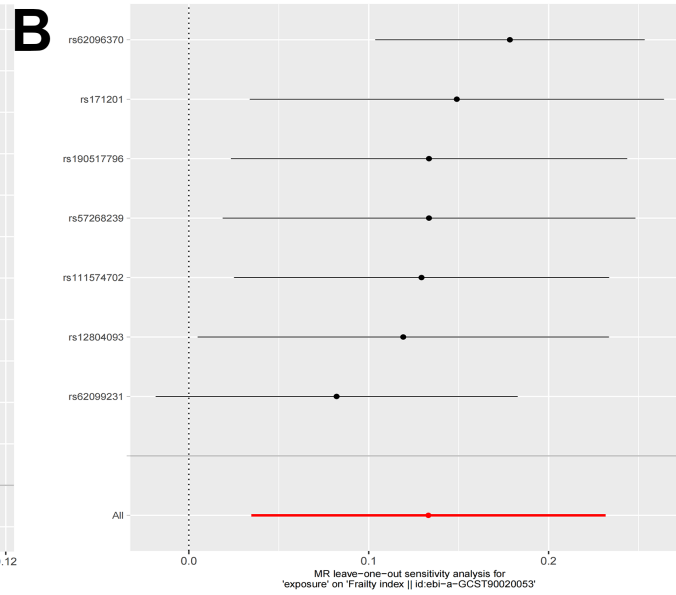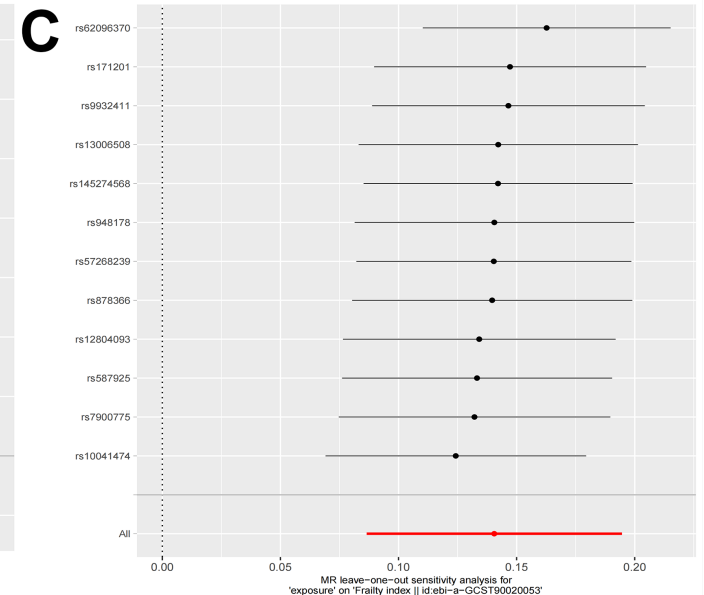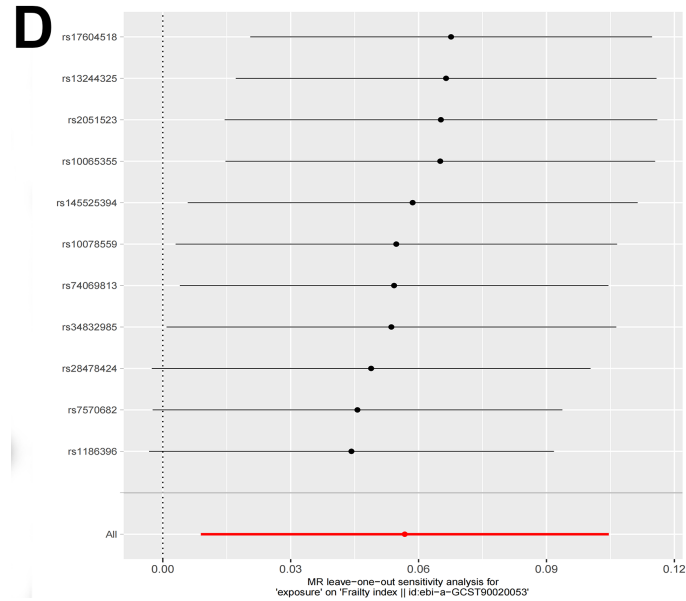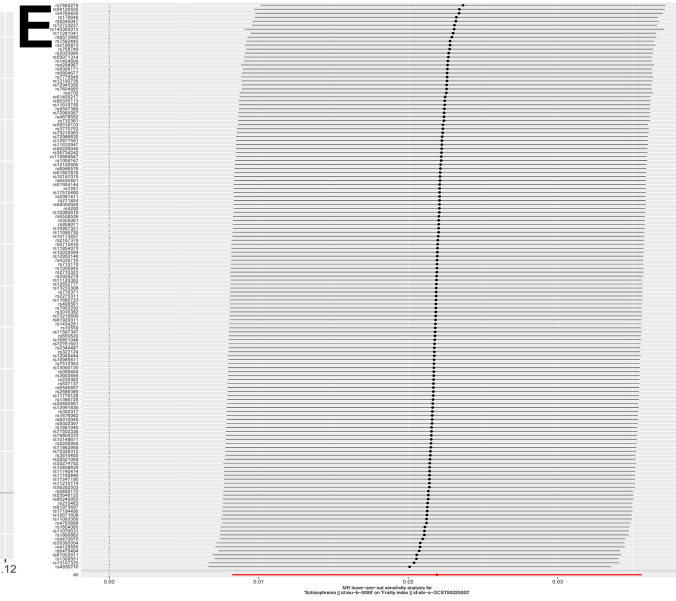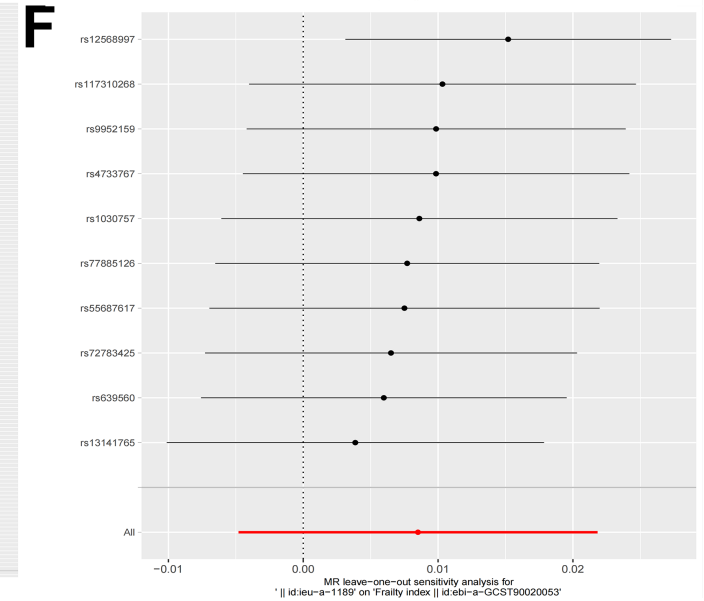

Supplement: Supplementary Figure 7 — Leave-one-out plots of two-sample Mendelian randomization analysis for genetically predicted anxiety (A), depression (B), affective disorder (C), mania (D), schizophrenia (E), and OCD (F) on FI outcomes. The dots indicate MR estimates for using inverse-variance weighted method when the SNP was removed. The bars indicate the confidence interval of MR estimates. [file Image_7.pdf]

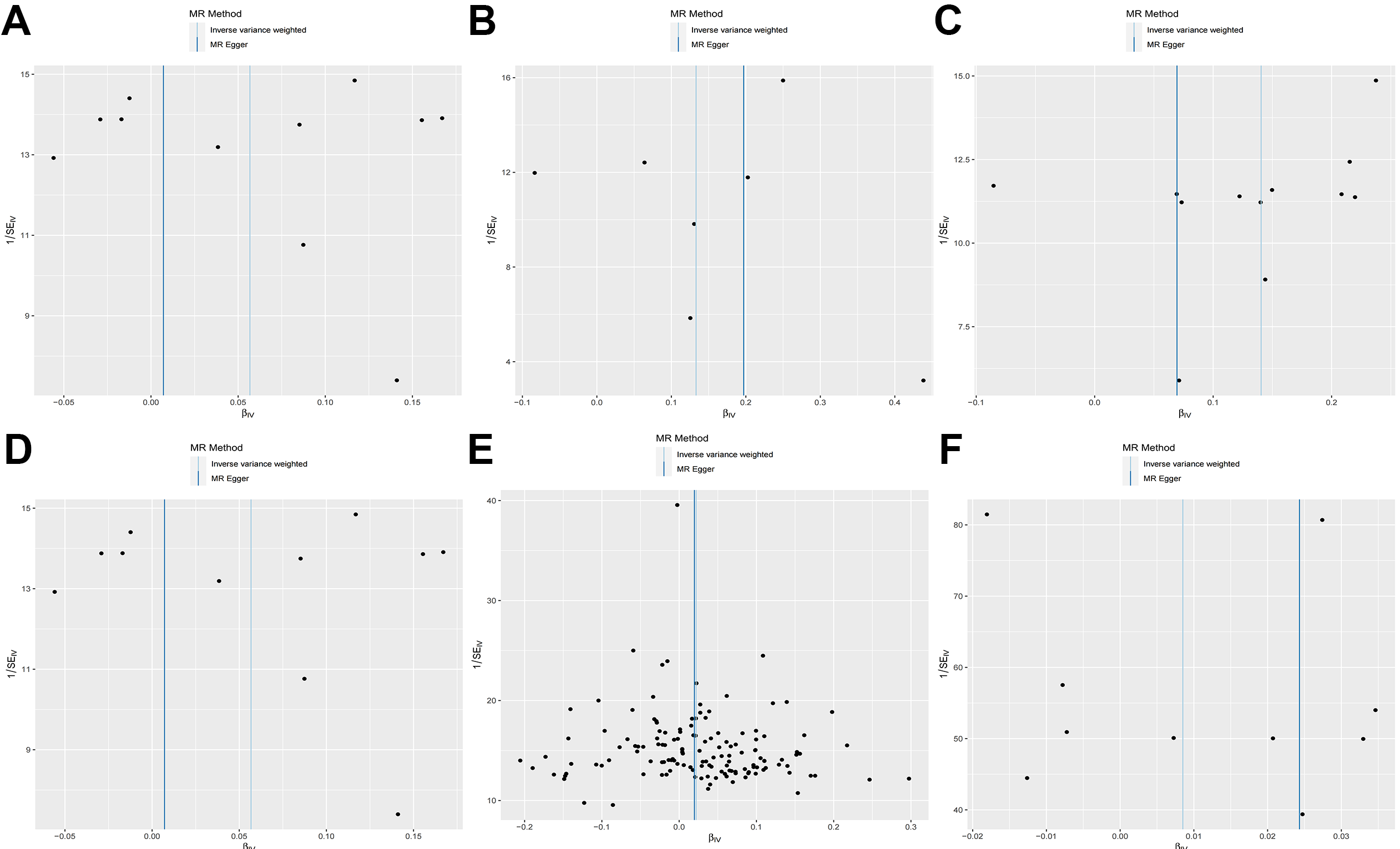

Supplement: Supplementary Figure 8 — Funnel plots assess the presence of potential heterogeneity across genetic instruments for anxiety (A), depression (B), affective disorder (C), mania (D), schizophrenia (E), and OCD (F) on FI, which indicates possible pleiotropic effects. The causal effect of each genetic instrument was presented by dots, and combined causal effect by inverse variance weighted and MR Egger were depicted by lines. [file Image_8.tif]
