# Supplementary material for: Bidirectional causal relational between frailty and mental illness: a two-sample Mendelian randomization study
Source: Front Psychiatry. 2024 Jun 7;15:1397813. doi: 10.3389/fpsyt.2024.1397813 (PMC11190300; doi:10.3389/fpsyt.2024.1397813)
Supplement: Supplementary file 9 [file DataSheet_1.docx]

| Exposure | SNP | Potential Confounders |
| --- | --- | --- |
| FI | rs2071207 | Alcohol intake frequency, alcohol intake vs 10 y previously, average weekly beer plus cider intake, ever highly irritable or argumentative for 2 days, fed-up feelings, miserableness, mood swings, sleeplessness or insomnia |
|  | rs82334 | Alcohol intake frequency |
|  | rs1363103 | Seen doctor for nerves, anxiety, tension or depression, sleeplessness or insomnia |
|  | rs2396766 | Insomnia, alcohol usually taken with meals, irritability, tense or highly strung |
|  | rs10891490 | Frequency of unenthusiasm or disinterest in the past 2 wk, guilty feelings, miserableness, neuroticism score, tense or highly strung |
| Anxiety | rs6795061 | Body fat percentage, trunk fat percentage, arm fat percentage, whole body fat mass, leg fat percentage, body mass index |
|  | rs743856 | Impedance of leg, impedance of whole body, impedance of arm, lung function (FVC), |
|  | rs10041968 | Lung function (FEV1/FVC) |
|  | rs10250881 | Forced vital capacity (FVC), overall health rating |
|  | rs641325 | Arm fat percentage, body fat percentage, heel bone mineral density, alcohol usually taken with meals |
|  | rs7317938 | Arm fat percentage, leg fat percentage, body fat percentage, body mass index, waist circumference |
| Depression | rs4619804 | Trunk fat percentage, body fat percentage, arm fat percentage, body fat percentage, whole body fat mass, leg fat percentage, |
|  | rs13086348 | Forced vital capacity, leg fat percentage, body mass index, body fat percentage, body mass index, arm fat percentage, hip circumference, waist-hip ratio, trunk fat percentage |
|  | rs3757323 | Forced vital capacity |
|  | rs1027190 | Alcohol intake frequency |
|  | rs7128734 | impedance of whole body, trunk fat-free mass, impedance of arm, impedance of leg, trunk fat-free mass |
|  | rs7192848 | Heel bone mineral density, forced vital capacity |
| Affective disorder | rs4619804 | Trunk fat percentage, Body fat percentage, Arm fat percentage, Leg fat percentage, Overall health rating, Body mass index, Whole body fat mass |
|  | rs699922 | Forced vital capacity, body mass index, leg fat percentage, waist-hip ratio, Arm fat percentage, trunk fat percentage, hip circumference, whole body fat mass, forced Expiratory Volume |
|  | rs76025409 | Body fat percentage, waist circumference |
|  | rs3757323 | Forced expiratory volume |
|  | rs7128734 | Smoking status, Impedance of whole body, impedance of arm, trunk fat-free mass, smoking initiation, whole body water mass, impedance of arm |
|  | rs12929749 | Heel bone mineral density, |
|  | rs62099231 | Forced vital capacity, overall health rating, alcohol intake frequency |
| mania | rs8180034 | Impedance of leg, impedance of whole body, impedance of arm, lung function |
|  | rs137891429 | Body mass index, arm fat percentage, arm fat mass |
| Obsessive compulsive disorder | rs12504244 | Body mass index, arm fat mass, hip circumference, waist circumference, leg fat mass, whole body fat-free mass, alcohol intake frequency, trunk fat-free mass |
|  | rs72781967 | Heel bone mineral density |
| Schizophrenia | rs10779702 | Bone mineral density, trunk fat percentage, impedance of leg ,body fat percentage, impedance of whole body, FEV1/FVC < 0.7 |
|  | rs12039854 | Trunk fat mass |
|  | rs61787564 | Trunk fat-free mass, whole body water mass, arm fat-free mass |
|  | rs2970610 | Alcohol intake versus 10 years previously |
|  | rs558120 | Impedance of whole body, impedance of arm, impedance of leg, |
|  | rs6588168 | Body mass index |
|  | rs4950119 | Heel bone mineral density, waist circumference, arm fat percentage, arm fat mass, whole body fat mass, body mass index, trunk fat mass, hip circumference, overall health rating |
|  | rs12138231 | Lung function, heel bone mineral density, |
|  | rs60124939 | Alcohol usually taken with meals |
|  | rs1486472 | body mass index, arm fat mass, arm fat percentage, |
|  | rs2139054 | Impedance of leg, impedance of whole body, alcohol intake frequency, waist-hip ratio, impedance of whole body |
|  | rs62152282 | Alcohol usually taken with meals |
|  | rs34181670 | Impedance of whole body, impedance of arm, trunk fat-free mass, arm fat-free mass, |
|  | rs60617652 | Impedance of arm, impedance of whole body, arm fat-free mass, impedance of leg, trunk fat-free mass, |
|  | rs10935184 | Waist-to-hip ratio adjusted for BMI, |
|  | rs525297 | Arm fat-free mass, whole body fat-free mass, waist circumference, arm fat mass, body mass index, impedance of whole body, trunk fat-free mass, leg fat-free mass, impedance of arm, hip circumference |
|  | rs7647398 | body mass index, leg fat mass, hip circumference, arm fat mass, |
|  | rs7681616 | Impedance of arm, body mass index, impedance of whole body, |
|  | rs10117 | Trunk fat-free mass, |
|  | rs7701188 | Bone mineral density, body mass index, heel bone mineral density |
|  | rs252812 | Impedance of arm, impedance of whole body, body mass index, impedance of leg, |
|  | rs3733710 | Arm fat-free mass, forced expiratory volume, whole body fat-free mass, arm predicted mass, hip circumference, forced vital capacity, trunk fat-free mass, body mass index |
|  | rs9687282 | Leg fat-free mass, whole body fat-free mass, hip circumference, trunk fat-free mass, peak expiratory flow, arm fat mass, impedance of leg |
|  | rs72802887 | Leg fat-free mass, |
|  | rs6938026 | Forced vital capacity, waist-hip ratio |
|  | rs9487653 | Trunk fat percentage, arm fat percentage, body fat percentage |
|  | rs7803571 | Impedance of arm, impedance of whole body, arm fat-free mass, trunk fat-free mass |
|  | rs1593304 | body mass index, waist circumference |
|  | rs2470951 | Whole body fat mass, trunk fat mass, leg fat mass, lung function, body fat percentage, waist circumference, leg fat percentage, arm fat mass, waist-hip ratio |
|  | rs73229090 | Alcohol intake versus 10 years previously, forced Expiratory Volume |
|  | rs59498392 | Impedance of leg, whole body water mass, leg fat-free mass, trunk fat-free mass, arm fat-free mass, |
|  | rs11987861 | Heel bone mineral density, impedance of leg, body mass index, arm fat mass, impedance of whole body, arm fat percentage |
|  | rs6471815 | Body mass index, waist circumference, arm fat mass |
|  | rs4043663 | Impedance of leg, impedance of whole body, impedance of arm, heel bone mineral density, waist-hip ratio |
|  | rs13293831 | Impedance of arm, arm fat-free mass |
|  | rs12571643 | Impedance of leg, leg fat-free mass, whole body fat-free mass, body mass index, trunk fat-free mass |
|  | rs17731 | Bone mineral density |
|  | rs708228 | Arm fat percentage, impedance of arm, trunk fat percentage, alcohol usually taken with meals |
|  | rs10767735 | Impedance of whole body, impedance of arm, trunk fat-free mass, alcohol usually taken with meals, smoking status, body mass index |
|  | rs2902858 | Bone mineral density, body mass index |
|  | rs11222406 | Arm fat mass, body mass index, arm fat percentage, whole body fat mass, trunk fat mass, hip circumference, waist circumference, impedance of leg |
|  | rs3017989 | Leg fat mass, waist circumference, arm fat mass, trunk fat mass, whole body fat mass, body mass index |
|  | rs578470 | Impedance of leg, impedance of whole body, impedance of arm |
|  | rs1790135 | Waist-hip ratio, heel bone mineral density, trunk fat-free mass, whole body fat-free mass, leg fat-free mass |
|  | rs1426371 | Leg fat percentage, body fat percentage, trunk fat percentage, impedance of leg, arm fat percentage, impedance of whole body, waist circumference, lung function |
|  | rs9569795 | Body mass index, arm fat mass, leg fat-free mass, waist circumference, impedance of whole body, hip circumference, Impedance of arm |
|  | rs12883788 | Body mass index, arm fat mass, whole body fat mass, trunk fat mass, body fat percentage, arm fat percentage, hip circumference, waist circumference, impedance of whole body |
|  | rs10873538 | Heel bone mineral density, impedance of leg, body mass index, waist circumference, smoking status, arm fat percentage |
|  | rs1540840 | Impedance of arm |
|  | rs9925915 | Whole body water mass, whole body fat-free mass, arm predicted mass, trunk fat-free mass, leg fat-free mass, arm fat-free mass, whole body fat-free mass, body mass index, hip circumference, waist circumference, impedance of leg |
|  | rs12925872 | Alcohol intake frequency, past tobacco smoking |
|  | rs8048039 | Waist-hip ratio |
|  | rs11647188 | Arm fat percentage, body mass index, trunk fat mass |
|  | rs11263770 | Body mass index, waist circumference, arm fat mass, leg fat mass, whole body fat mass, trunk fat mass |
|  | rs12943566 | Heel bone mineral density, impedance of leg, impedance of whole body, lung function, body mass index, impedance of arm |
|  | rs7238071 | Overall health rating |
|  | rs8101499 | Impedance of leg, impedance of whole body |
